# Supplementary material for: tDCS modulates effective connectivity during motor command following; a potential therapeutic target for disorders of consciousness
Source: Neuroimage. 2022 Feb 15;247:118781. doi: 10.1016/j.neuroimage.2021.118781 (PMC8803542; doi:10.1016/j.neuroimage.2021.118781)
Supplement: Supplementary file 1 [file mmc1.docx]

**Supplementary Material**

**Table S1. Effect of M1-tDCS on brain activation.**

| **Contrast** | **Region** | **Cluster P** | **Cluster size** | **Peak P** | | **F / T** | **MNI coordinates** |
| --- | --- | --- | --- | --- | --- | --- | --- |
|  |  | *FWE-corrected* | *in mm3* | *FWE-corrected* | *uncorrected* |  | *[x;y;z]* |
| ***Interaction between polarity and time*** | *Thalamus* | 0.967 | 54 | 0.965 | <0.001 | 8.092 | [-3;-16;-1] |
| ***Greater Increase after anodal as compared to cathodal*** | *SMA* | 0.886 | 108 | 0.790 | <0.001 | 3.550 | [-9;11;53] |
|  |  | 0.935 | 54 | 0.907 | <0.001 | 3.383 | [-9;20;56] |
| ***Greater increase after cathodal as compared to sham*** | *Thalamus* | 0.935 | 54 | 0.443 | <0.0001 | 3.901 | [-3;-16;-1] |
| ***Greater increase after anodal as compared to sham*‡** | *Thalamus* | 0.999 | 189 | 0.993 | 0.001 | 3.069 | [-15;-25;-1] |
|  |  | 0.998 | 216 | 0.999 | 0.002 | 2.868 | [-6;-13;-1] |

Results from the random effect group analyses on the brain activation during thumb movements to command. We include results that survive a threshold of p<0.001 uncorrected (p<0.005 in the case of **‡**). In addition, we do not include spurious single voxel activations. Abbreviations: FWE, family wise error; MNI, Montreal Neurological Institute; SMA, supplementary motor area.

Table S2. Effects of tDCS on behavioural metrics

| **Metrics** | **Polarity** | **Baseline** | **Post-tDCS** | **t _(baseline vs post-tDCS)_** | **p-Holm**  **_(baseline vs post-tDCS)_** | **F _(main effect time)_** | **p _(main effect time)_** | **F _(interaction)_** | **p _(interaction)_** |
| --- | --- | --- | --- | --- | --- | --- | --- | --- | --- |
| ***Experiment 1 - M1-tDCS*** | | | | | | | | | |
| **Reaction time (**$\boldsymbol{s}$**)** | **Anodal** | 0.29 (± 0.06) | 0.27 (± 0.05) | 2.389 | 0.314 | 3.990 | 0.063 | 1.312 | 0.283 |
|  | **Cathodal** | 0.27 (± 0.05) | 0.26 (± 0.04) | 0.626 | 1.0 |  |  |  |  |
|  | **Sham** | 0.28 (± 0.05) | 0.28 (± 0.07) | 0.102 | 1.0 |  |  |  |  |
| **Mean Velocity**  **(**$\boldsymbol{cm/s}$**)** | **Anodal** | 8.39 (± 3.98) | 7.59 (± 2.81) | 2.697 | 0.145 | 04.430 | 0.051 | 1.939 | 0.160 |
|  | **Cathodal** | 7.71 (± 4.49) | 7.26 (± 3.83) | 0.868 | 1.0 |  |  |  |  |
|  | **Sham** | 6.96 (± 3.14) | 6.99 (± 2.83) | 0.120 | 1.0 |  |  |  |  |
| **Peak acceleration**  **(**$\boldsymbol{m/}\boldsymbol{s}^{\boldsymbol{2}}$**)** | **Anodal** | 31.05 (± 41.64) | 37.31 (± 56.13) | 0.312 | 1.0 | 0.616 | 0.444 | 0.636 | 0.536 |
|  | **Cathodal** | 32.81 (± 38.32) | 24.11(± 29.34) | 0.325 | 1.0 |  |  |  |  |
|  | **Sham** | 77.82 (± 177.01) | 111.39 (± 244.91) | 1.299 | 1.0 |  |  |  |  |
| ***Experiment 2 - cb-tDCS*** | | | | | | | | | |
| **Reaction time (**$\boldsymbol{s}$**)** | **Anodal** | 0.30 (± 0.04) | 0.28 (± 0.04 ) | 3.669 | 0.008** | 21.094 | <0.001** | 0.951 | 0.395 |
|  | **Cathodal** | 0.29 (±0.03) | 0.28 (± 0.04) | 1.998 | 0.601 |  |  |  |  |
|  | **Sham** | 0.30 (± 0.05) | 0.29 (±0.05) | 1.885 | 0.601 |  |  |  |  |
| **Mean Velocity**  **(**$\boldsymbol{cm/s}$**)** | **Anodal** | 7.44 (± 3.53) | 7.78 (± 3.98) | 0.626 | 1.0 | 0.286 | 0.598 | 1.106 | 0.340 |
|  | **Cathodal** | 7.11 (± 3.18) | 6.38 (± 2.77) | -1.486 | 1.0 |  |  |  |  |
|  | **Sham** | 8.09 (± 2.73) | 8.09 (± 2.55) | -0.007 | 1.0 |  |  |  |  |
| **Peak acceleration**  **(**$\boldsymbol{m/}\boldsymbol{s}^{\boldsymbol{2}}$**)** | **Anodal** | 39.29 (± 31.94) | 39.29 (± 33.62) | 0.001 | 1.0 | 1.823 | 0.191 | 0.404 | 0.670 |
|  | **Cathodal** | 51.19 (± 96.90) | 31.74 (± 15.27) | -0.372 | 1.0 |  |  |  |  |
|  | **Sham** | 77.82 (± 141.91) | 60.96 (± 108.44) | -1.046 | 1.0 |  |  |  |  |

Statistics for the post hoc (baseline vs post-tDCS) tests, main effect of time, and interaction between polarity (anodal, cathodal, sham) and time (baseline vs post-tDCS) on average reaction time, mean velocity and peak acceleration for Experiment 1 and Experiment 2. p-Holm value adjusted for comparing a family of 15; p_(interaction)_ uncorrected; **p<.01. Abbreviations: ms, milliseconds; cm, centimetres; s, seconds; m, metres.

**Table S3. Blinding**

| **Experiment** | **Active Stimulation** | **Sham** | $\boldsymbol{\chi}\boldsymbol{2}$ | **p** |
| --- | --- | --- | --- | --- |
| ***Experiment 1*** | 31/43 | 15/22 | 0.9682 | 0.701 |
| ***Experiment 2*** | 33/40 | 13/22 | 0.0869 | 0.263 |

Number of times that each type of stimulation was perceived as real, and statistics for the corresponding McNemar’s Test. Active stimulation includes anodal and cathodal sessions.

**Figure S1.**

**
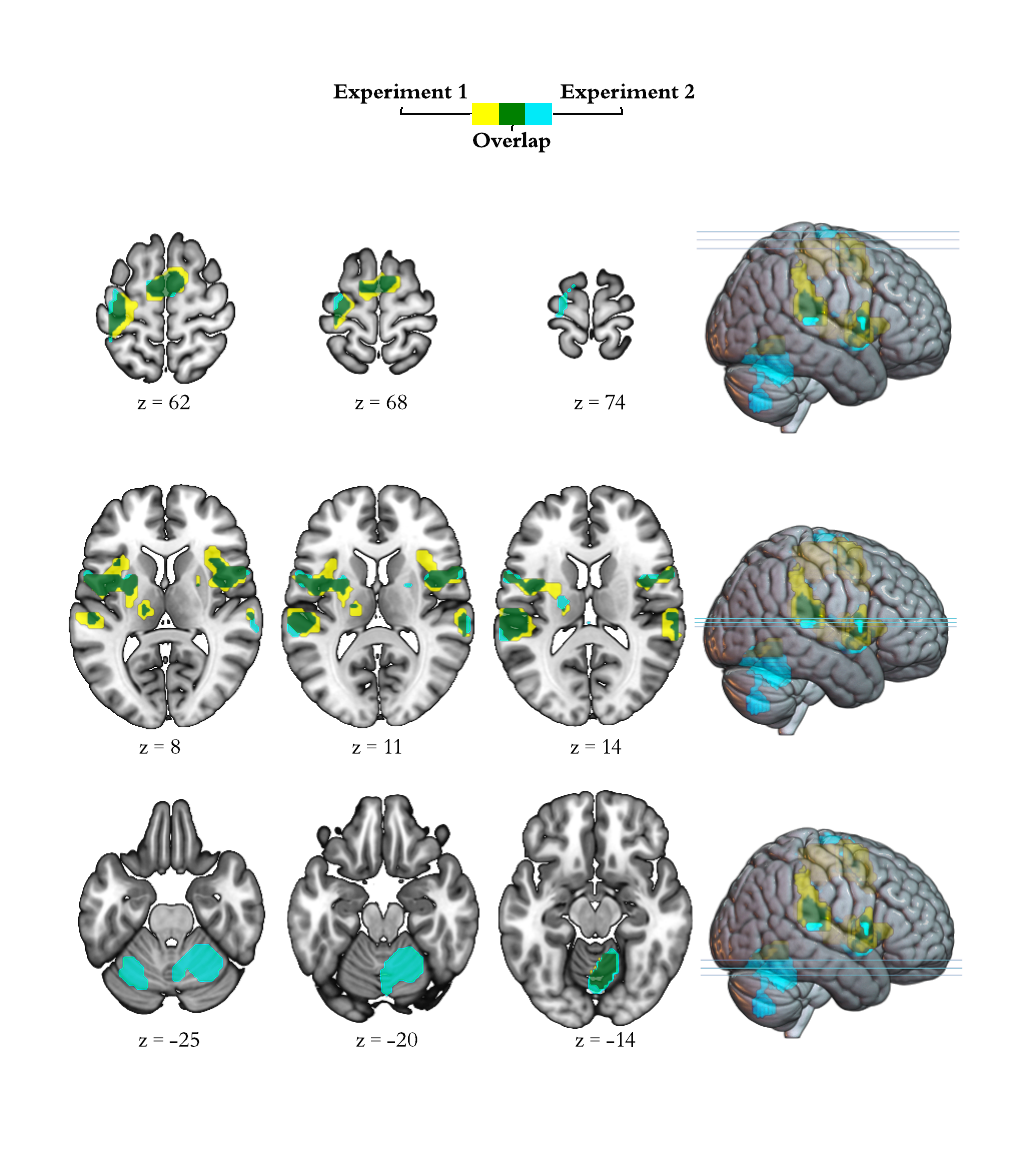
**

**Brain activation during command following across trials.**

The insets display group general linear model differences between ‘move’ and ‘rest’ blocks in Experiment 1 (yellow) and Experiment 2 (light blue), across all trials included in the ANOVA (positive effect of task). The overlap across experiments appears in green. Activation maps are shown at a FWE p<0.05 and rendered on a standard template (152 template in MRIcroGL). We display whole brain results as per request during peer review. z indicates the Montreal Neurological Institute z coordinate.
